# Comparison of qRT-PCR and ddPCR for multi-strain probiotic detection after a randomized human clinical trial

**DOI:** 10.3389/fmicb.2025.1579797

**Published:** 2025-04-28

**Authors:** Nicolas Yeung, Arthur C. Ouwehand

**Affiliations:** Global R&D, IFF Health Sciences, Kantvik, Finland

**Keywords:** probiotic quantification, probiotic detection, clinical trial, *Lactobacillus*, *Bifidobacterium*, Ncfm, Bl-04, Lpc-37

## Abstract

The ability to detect probiotic consumption during a human clinical trial is crucial to verify and validate placebo and verum groups in *post hoc* analysis. While bacterial plating is still a common method for detecting and counting bacteria, when dealing with complex matrices like fecal samples, and given that most probiotics share genera or even species with commensal bacteria, plate counting is not a precise or accurate enough method. Species-specific quantitative real-time polymerase chain reaction (qRT-PCR) has been the most cited method in the literature and when properly validated and optimized remains the high watermark for detecting probiotics from fecal samples. Recent advancements in PCR technology have given rise to a parallel platform, droplet digital PCR (ddPCR). In this work we aimed to detect the components of a multi-strain probiotic product from a human clinical trial and compare both methods. This work dually demonstrates a process for determining multi-strain detection criteria as well as directly comparing the methods through the lens of sensitivity and specificity or the ability to properly discern true positives and true negatives. We described the optimization and validation of three assays for use in our detection panel and observed that, between qRT-PCR and ddPCR. The two methods were found to be quite congruent with ddPCR demonstrating a 10–100 fold lower limit of detection. Moreover, we discovered that most of the sensitivity and specificity had come from a single assay alone (*Bifidobacterium animalis* subsp. *lactis* Bl-04). This is despite all three assays performing well in optimization and validation. This suggests that more work needs to be done in the validation stage when developing novel probiotic detection assays. Taken together we can recommend ddPCR as a method for detecting probiotics from human clinical trials, but that qRT-PCR still performs well and comparably to ddPCR, when properly optimized and validated. However, when novel assays or those with unknown performance in a given biological matrix are needed, employing a strategy that combines multiple assays in a layered discrimination approach can help mitigate the potential underperformance of any single assay.

## Introduction

Clinical trials are the keystone for demonstrating the health benefits associated with probiotic use and are an intrinsic part of the definition of probiotics: “live organisms that, when administered in adequate amounts, confer a health benefit on the host” (Hill et al., 2014). The ability to detect a probiotic after consumption and digestion is a crucial step in validating the treated (verum) and untreated (placebo) groups, after a clinical trial. Given that probiotic health benefits are assigned at the strain-level the method of choice for most researchers has been quantitative real-time polymerase chain-reaction (qRT-PCR). This method requires appropriate assay design, optimization, and validation to be able to detect minute genomic differences in the hopes of discerning one strain from another, and in most cases can be done successfully. PCR, as a technology, has evolved through the years and a new generation of PCR, digital PCR (dPCR), has the potential to supersede qRT-PCR in this application (Taylor et al., 2017; Hansen et al., 2019). The fundamental idea of digital PCR is to sub-partition a PCR assay into tens of thousands of individual compartments that each contain the constituents for a PCR reaction (Sykes et al., 1992). Then, based on the number of detected events a Poisson correction (correcting for the likelihood of multiple copies in one partition) can be applied to determine the starting number of targets, essentially quantifying without the need for a standard curve. In addition to this advantage of dPCR compared with qRT-PCR, there are demonstrated increases in: precision (Sanders et al., 2011), dynamic range (Whale et al., 2012), and a reduced susceptibility to carryover of PCR-inhibitors (Hoshino and Inagaki, 2012). In addition, since both methods are based on PCR, the assay primer design is the same for both approaches. Meaning that the designed primers can be transferred from qRT-PCR to dPCR, with the need for additional validation and optimization on the dPCR platform. The various instruments that can perform dPCR vary in their strategies for the crucial step of sub-partitioning. Advancements in plastics manufacturing have allowed microfluidic channels to be etched into microtiter plate cartridges. These channels allow nanoliter sized micelle droplets to be created by a hydrophobic oil surrounding and partitioning the aqueous PCR reaction constituents. This method is referred to as droplet dPCR (ddPCR) (Hindson et al., 2011).

In a recent clinical trial (Airaksinen et al., 2019), which investigated the effects of a multi-strain probiotic on gastrointestinal symptoms of constipated subjects, qRT-PCR was used to monitor compliance. The multi-strain blend consisted of 5 strains: *Lactobacillus acidophilus* NCFM (NCFM), *Lacticaseibacillus paracasei* Lpc-37 (Lpc-37), *Bifidobacterium animalis* subsp. *lactis* Bl-04 (Bl-04), *B. lactis* Bi-07 (Bi-07) and *B. lactis* HN019 (HN019). qRT-PCR assays targeting three strains (NCFM, Lpc-37, and Bl-04) were reported for detecting probiotic consumption. The assays used (Table 1) were simultaneously optimized for ddPCR, and available samples were also run using ddPCR. This approach of targeting three out of five of the strains was taken due to the difficulty, at the time, of designing PCR primers and probes to detect the members of the *B. lactis* species due to their highly similar genomes (Milani et al., 2013).

**Table 1:** PCR Primers and Probes used with sequence information and concentrations, for both qRT-PCR and ddPCR, and annealing temperatures used in each assay along with their source references.

Target Species	Name	Sequence 5′ to 3′	Concentration (μM)	Annealing Temperature (°C)	Reference
*Lactobacillus acidophilus* NCFM	Laci_NCFMMJ_RTfwd	CCACGACCAGATGTAACCAA	200	62	Airaksinen et al. (2019)
	Laci_NCFM_Rtrev	TTAGAAGATGCCAACGTCGAG	600		
	Laci_NCFM_probe	HEX-TAAGCCGAA/ZEN/CAATGCTGAAACGAT-IABkFQ	900		
*Lacticaseibacillus paracasei* Lpc-37	F_paca_IS	ACATCAGTGTATTGCTTGTCAGTGAATAC	240	60	Haarman and Knol (2006)
	R_paca_IS	CCTGCGGGTACTGAGATGTTTC	240		
	P_paca_IS	TGCCGCCGGCCAG	400		
*Bifidobacterium animalis* subsp*. lactis* Bl-04	Bl04_for	CTTCCCAGAAGGCCGGGT	100	60	Lehtinen et al. (2018)
	Bl04_rev	CGAGGCCACGGTGCTCATATAGA	100		

This work aimed to compare the qRT-PCR and ddPCR across a human clinical trial sample set. To that end, each assay was measured in terms of its “sensitivity” or true positive rate (an assays ability to detect true positives, samples from the treated group), and its “specificity” or true negative rate (and assays ability to detect true negatives, samples from the untreated group), which are described in detail in the materials and methods (Yerushalmy, 1947; Glaros and Kline, 1988). This kind of analysis is not possible during a clinical trial, as it requires beforehand knowledge of the treatment groups which is usually blinded during the clinical trial but can serve as a useful diagnostic tool for evaluating the effectiveness of a given assay after a trial. In Airaksinen et al., 2019 a criterion for multi-strain probiotic detection by qRT-PCR was set as any sample being positive for more than one assay. Here we will look at the assays individually and, in all combinations greater than one to map how this criterion performed in its goal of discriminating the treated from untreated groups across both qRT-PCR and ddPCR.

## Materials and methods

### Data set

**Figure 1:**
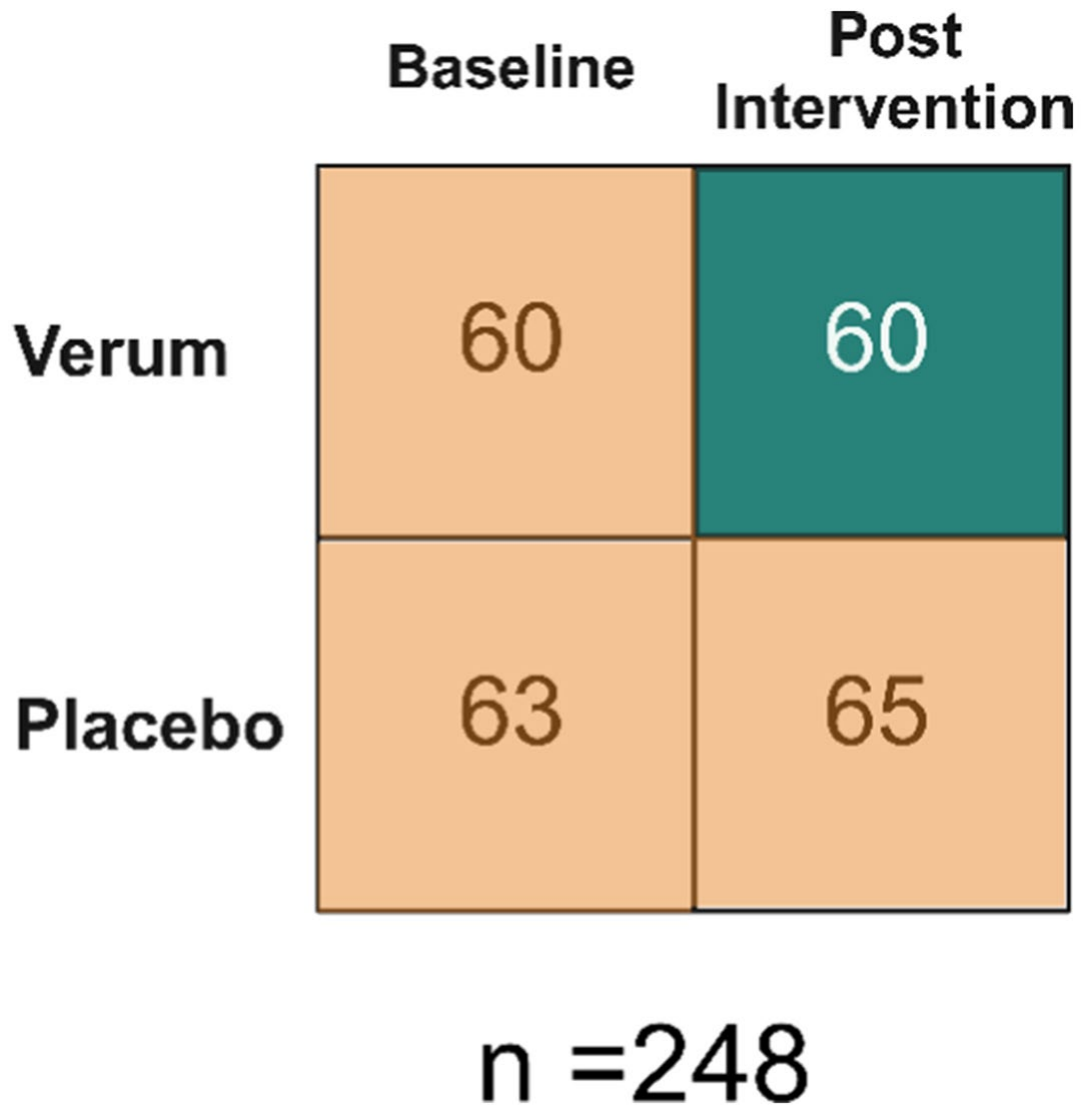
Data Set Overview. Baseline refers to prior to any intervention. Therefore, there are 60 samples that make up the true positives and 188 that make up the true negatives.

The data set consisted of 248 samples split by two dimensions: baseline/post intervention and verum/placebo (Figure 1). There were 60 samples in the verum and post intervention category (i.e., true positive/treated group), and there were 188 samples combined from: baseline verum, baseline placebo and post intervention placebo (i.e., true negatives/untreated group). The samples were collected from the study reported by Airaksinen et al., 2019. Constipated adults consumed a total of 2.75 × 10^10^ CFU/day consisting of a combination of 5 probiotic strains (*L. acidophilus* NCFM (10^10^ CFU), *L. paracasei* Lpc-37 (2.5×10^9^ CFU), *B. lactis* Bl-04 (2.5 × 10^9^ CFU), *B. lactis* Bi-07 (2.5 × 10^9^ CFU) and *B. lactis* HN019 (10^10^ CFU)) or placebo for 2 weeks. The sample set is different from what was reported in Airaksinen et al., 2019 as the priority for the trial was the qRT-PCR data and not all samples were of sufficient amount to re-run on ddPCR. The data set is asymmetrical because paired subject samples (baseline and post-intervention) were not the primary focus of this analysis; instead, emphasis was placed on comparing two methods.

### DNA isolation and quantification

DNA was extracted as described in Airaksinen et al., 2019, in brief: 200 mg of fecal sample (stored at −80°C immediately after collection and during transportation) was extracted by using the AM1840 MagMax™ Total Nucleic Acid Isolation kit on the MagMax™ Express 96 Magnetic Particle Processor (Thermo Fisher Scientific, Vantaa, Finland). After being weighed, but prior to MagMax™ DNA isolation, samples were lysed using the lysis/binding buffer from the AM1840 kit and bead beating using the Precellys VK01 bead tubes and beaten using Precellys 24 Tissue Homogenizer (2 cycles of 3 pulses for 30 s at 6800 RPM) (Bertin Technologies, Montigny-le-Bretonneux, France). DNA was quantified using the Qubit HS kit on the Qubit 3.0 Fluorimeter (Thermo Fisher). Pure culture bacterial DNA (for standard curves and assay optimization) was extracted in the same manner but starting from 1 mL of bacterial culture.

### qRT-PCR

qRT-PCR was performed as described in Airaksinen et al., 2019, but briefly: all qPCR assays were run on the 7500FAST Real-Time PCR Systems and either SYBR Fast or Taqman Fast Advanced mastermixes (Applied Biosystems, Waltham, MA, USA). All primers and probes were produced by Integrated DNA Technologies IDT (Coralville, IA, USA), sequences and annealing temperatures are listed in Table 1. Assay conditions were optimized using a primer concentration matrix and temperature gradient. qRT-PCR reactions, from clinical trial samples, were run with 10 ng of isolated fecal DNA. Each assay was run individually and not multiplexed.

### ddPCR

ddPCR was performed using the Bio-Rad Laboratories (Hercules, CA, USA) QX200™ family of instruments and reagents: QX200 Droplet Reader, Automated Droplet Generator, ddPCR Supermixes either EvaGreen or for Probes (No dUTP). All primers and probes were produced by Integrated DNA Technologies IDT, sequences and annealing temperatures are listed in Table 1. ddPCR reactions, from clinical trial samples, were run with 10 ng of isolated fecal DNA. Fluorescence intensity thresholds were properly set, with minimal rain observed, and a minimum of 10^5^ droplets were observed for any sample quantification. Each assay was run individually and not multiplexed.

### Sensitivity and specificity

We applied the metrics of “sensitivity” (true positive rate) and “specificity” (true negative rate) to evaluate the performance of the diagnostic assays (Equation 1) (Yerushalmy, 1947; Glaros and Kline, 1988). In other words, sensitivity is the ability of an assay to properly observe a positive signal from a known positive sample and specificity is the ability for the same assay not to observe a signal from a known negative sample. The inverse concepts of these ideas can be inferred from these ratios (i.e., if an assay has a sensitivity of 80%, it also missed 20% of the positive samples or false negatives, etc.).


$$\begin{array}{l} \mathrm{Sensitivity}\%=\\ \frac{\mathrm{Samples}\;\mathrm{positive}\;for\;\mathrm{an}\;\mathrm{assay}}{\mathrm{Number}\;\mathrm{of}\;\mathrm{samples}\;\mathrm{in}\;\mathrm{the}\;\mathrm{treated}\;\mathrm{group}\;\left(n=60\right)}\;x\;100\% \end{array}$$

$$\begin{array}{l} \mathrm{Specificity}\%=\\ \frac{\mathrm{Samples}\;\mathrm{negative}\;for\;\mathrm{an}\;\mathrm{assay}}{\mathrm{Number}\;\mathrm{of}\;\mathrm{samples}\;\mathrm{in}\;\mathrm{the}\;\mathrm{untreated}\;\mathrm{group}\;\left(n=188\right)}\;x\;100\% \end{array}$$

Equation 1: the definition for both sensitivity and specificity used in this study.

### Statistics

Raw data was exported from proprietary instruments’ software by Microsoft Excel (Excel) files. Data management was done using RStudio 2024.04.2 Build 764 R version 4.4.1. Sensitivity and specificity were calculated in Excel and correlations were done using R package Corrplot v0.9.2 with Pearson correlation for qRT-PCR v ddPCR (Wei and Simko, 2021).

## Results

### Assay optimization, validation and raw data

**Figure 2:**
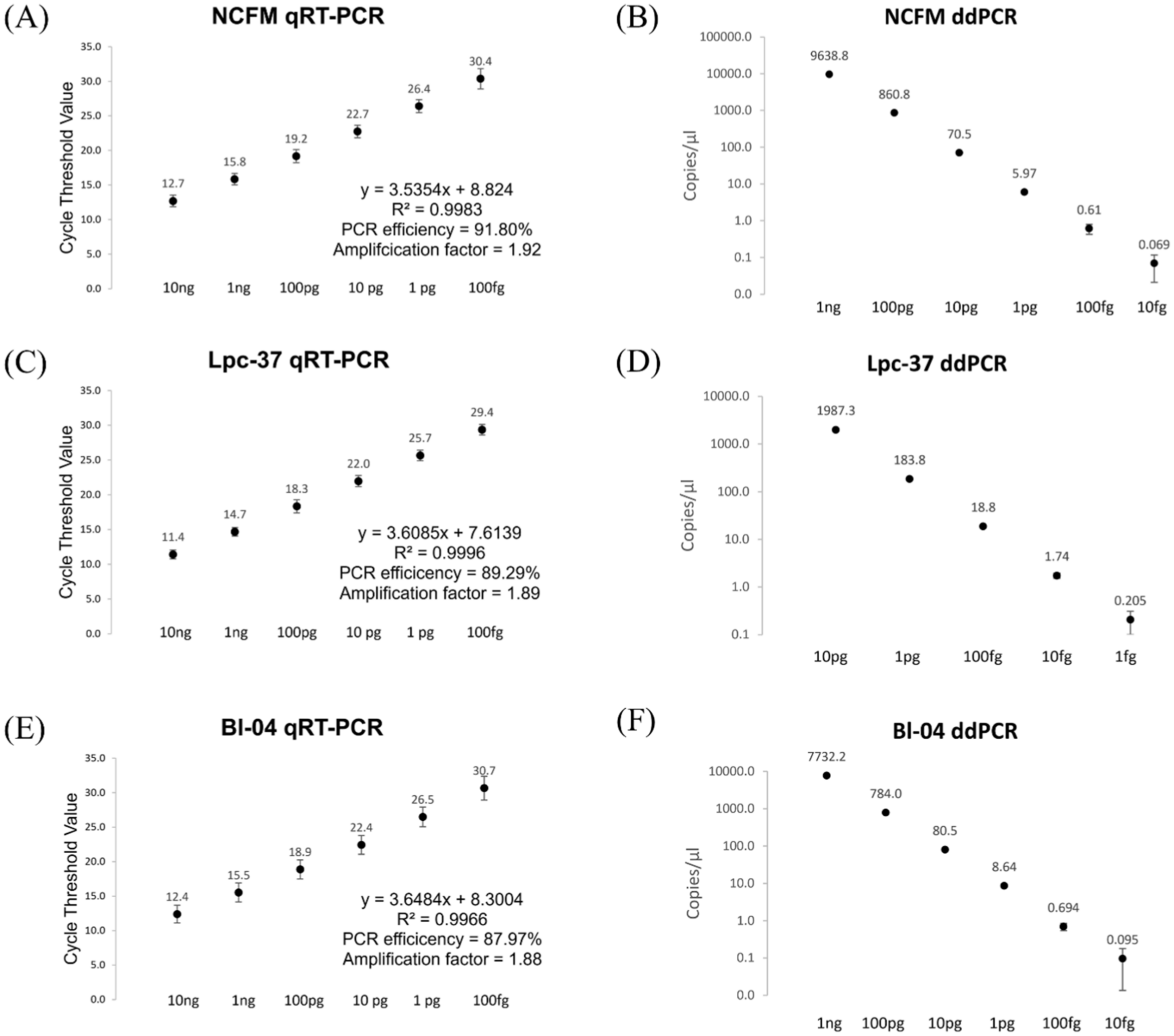
qRT-PCR, panels **(A,C,E)** and ddPCR, Panels **(B,D,F)** assay performance from a standard curve of pure culture bacterial DNA. Averages with standard deviations shown. In panels **(A,C,E)** PCR efficiency was calculated using a slope of −3.322 as 100% PCR efficiency. In panels **(B,D,F)** the copies/μl scale is logarithmic with a base of 10.

To evaluate the performance of the assays, prior to use as a diagnostic tool, we performed several optimization and validation experiments. The three assays were evaluated for PCR efficiency by applying them to a standard curve of pure culture bacterial DNA, for their respective target strains (Figure 2). The optimal assay conditions (primer and probe concentrations, and annealing temperature) were found to be the same for qRT-PCR and ddPCR. The qRT-PCR assays all performed consistently (Figure 2: panels A, C, E) along a dynamic range of 10 ng to 100 fg of bacterial DNA with an average PCR efficiency of 89.69%. ddPCR assays also performed linearly along a descending 10-fold dilution series, (Figure 2: panels B, D, F). ddPCR demonstrated a lower limit of detection as compared to qRT-PCR, 10 fg compared to 100 fg, respectively, (except for Lpc-37 which showed a lower limit of detection with ddPCR at 1 fg).

The assays were evaluated for off target detection using a panel of bacterial strains and a commercially available mock microbial community, from Zymo Research (Irvine, CA, USA) (Table S1). The NCFM assay detected *Lactobacillus acidophilus* strain La4356 but not La-14. The Lpc-37 assay was able to distinguish between *Lacticaseibacillus* species *paracasei* and *casei*. The Bl-04 assay detected *Bifidobacterium animalis* subsp. *lactis* Bi-07 but not strains HN019 or B420. All three assays apart from these exceptions described, were designed well enough to not detect other bacteria from their respective panels.

The optimized and validated assays were then applied to the DNA extracted from human fecal samples. The descriptive statistics for the absolute values from both methods across all assays can be seen in Table 2. There was no outlier removal in this analysis even though in qRT-PCR and ddPCR some of the strongest signals came from the true negative group. This reflects the blinded nature of the analysis that was done during the clinical trial as the participants groups are not visible to those performing or analyzing the results.

**Table 2:** Descriptive statistics of qRT-PCR and ddPCR for all assays with lower limits of quantification (LLOQ).

Data sets	Statistical indicators	NCFM		Lpc-37		Bl-04	
		qRT-PCR [genomes/10 ng]	ddPCR [copies/μl]	qRT-PCR [genomes/10 ng]	ddPCR [copies/μl]	qRT-PCR [genomes/10 ng]	ddPCR [copies/μl]
LLOQ (Lower Limit of Quantification)		100 fg	10 fg	100 fg	1 fg	100 fg	10 fg
		46.2	0.07	27.8	0.2	47.5	0.1
All Data (*n* = 248)	Min	24.9	0.5	20.7	0.3	26.2	0.4
	Max	155335.9	1767.0	31026.4	2564.0	219137.8	122.6
	Mean	10407.5	107.0	1160.6	123.7	3836.4	13.0
	Std. Dev.	29203.9	301.6	2914.7	284.4	26515.4	20.1
	*n*	56	40	141	194	67	50
True Positives (*n* = 60)	Min	29.3	0.5	21.7	0.4	26.2	0.4
	Max	10063.5	45.7	11879.3	2050.0	4991.7	122.6
	Mean	1159.5	7.7	1172.9	181.1	680.2	13.9
	Std. Dev.	2134.9	11.7	1698.5	295.9	933.4	20.9
	*n*	24	19	56	57	49	45
True Negatives (*n* = 188)	Min	24.9	0.6	20.7	0.3	27.5	0.7
	Max	155335.9	1767.0	31026.4	2564.0	219137.8	17.0
	Mean	17343.5	196.8	1152.5	99.8	12428.2	5.5
	Std. Dev.	37105.9	395.1	3491.6	276.0	50136.4	6.2
	*n*	32	21	85	137	18	5

Assays were quantifiable above the LLOQ and below this, assigned a value of half the LLOQ.

### Single assay sensitivity and specificity

**Figure 3:**
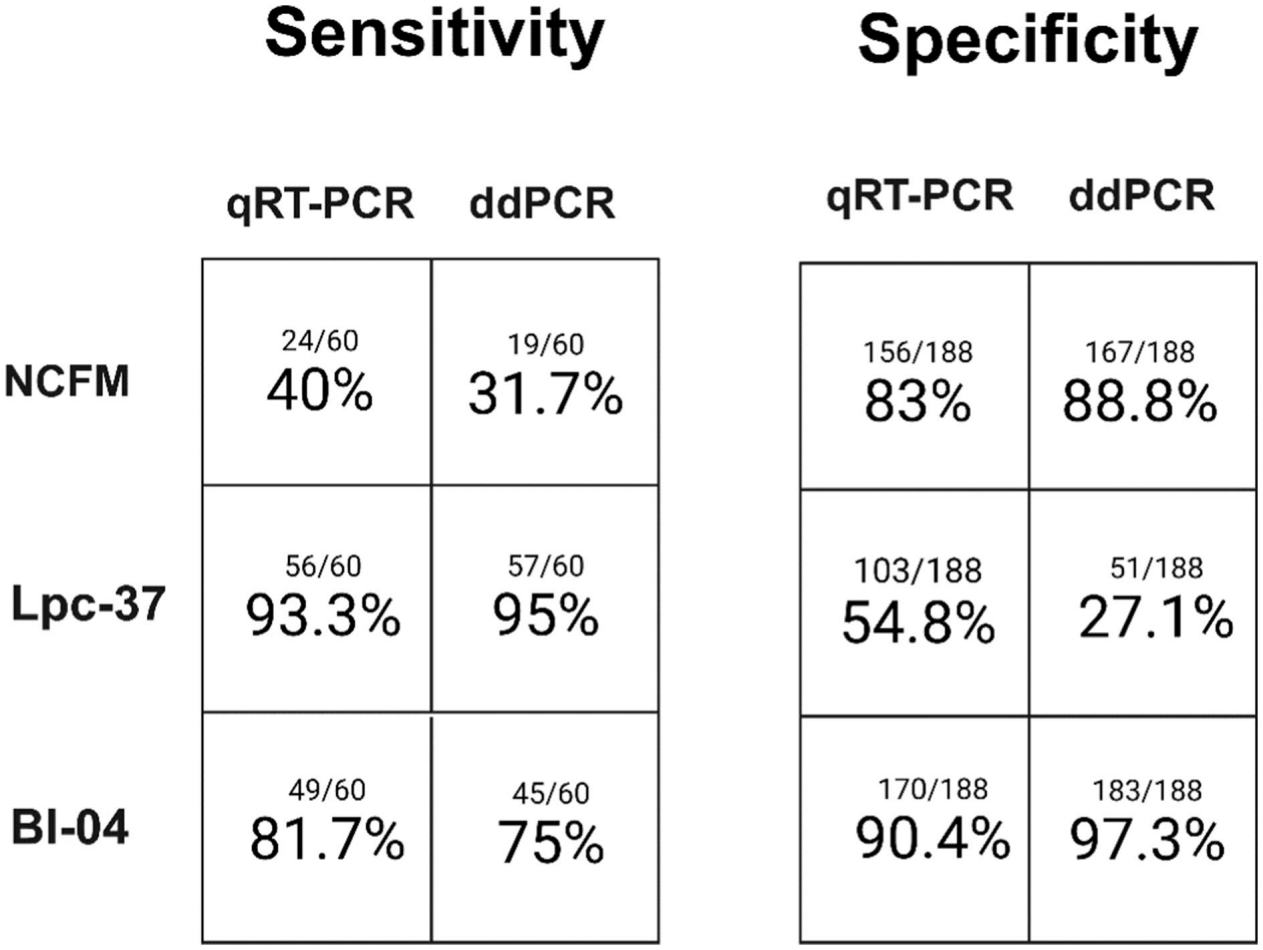
Single assay sensitivity (samples observed positive for an assay divided by total samples expected to be positive) and specificity (samples observed to be negative for an assay divided by total samples expected to be negative) for both qRT-PCR and ddPCR.

Each assay was individually assessed across the dimensions of sensitivity and specificity between qRT-PCR and ddPCR (Figure 3). Each assay performed similarly between qRT-PCR and ddPCR in terms of both sensitivity and specificity. The NCFM assay had the lowest sensitivity in both qRT-PCR (40%) and ddPCR (31.7%) while the Lpc-37 assay had the lowest specificity in both qRT-PCR (54.8%) and ddPCR (27.1%). However, the Lpc-37 assay while having the lowest specificity also demonstrated the highest sensitivity in both qRT-PCR (93.3%) and ddPCR (95%), highlighting the interplay of the two concepts and the trade-off when trying to maximize both. Overall, the Bl-04 assay performed the best in terms of both sensitivity (qRT-PCR 81.7%, ddPCR 75%) and specificity (qRT-PCR 90.4%, ddPCR 97.3%).

### Multi-assay sensitivity and specificity

**Figure 4:**
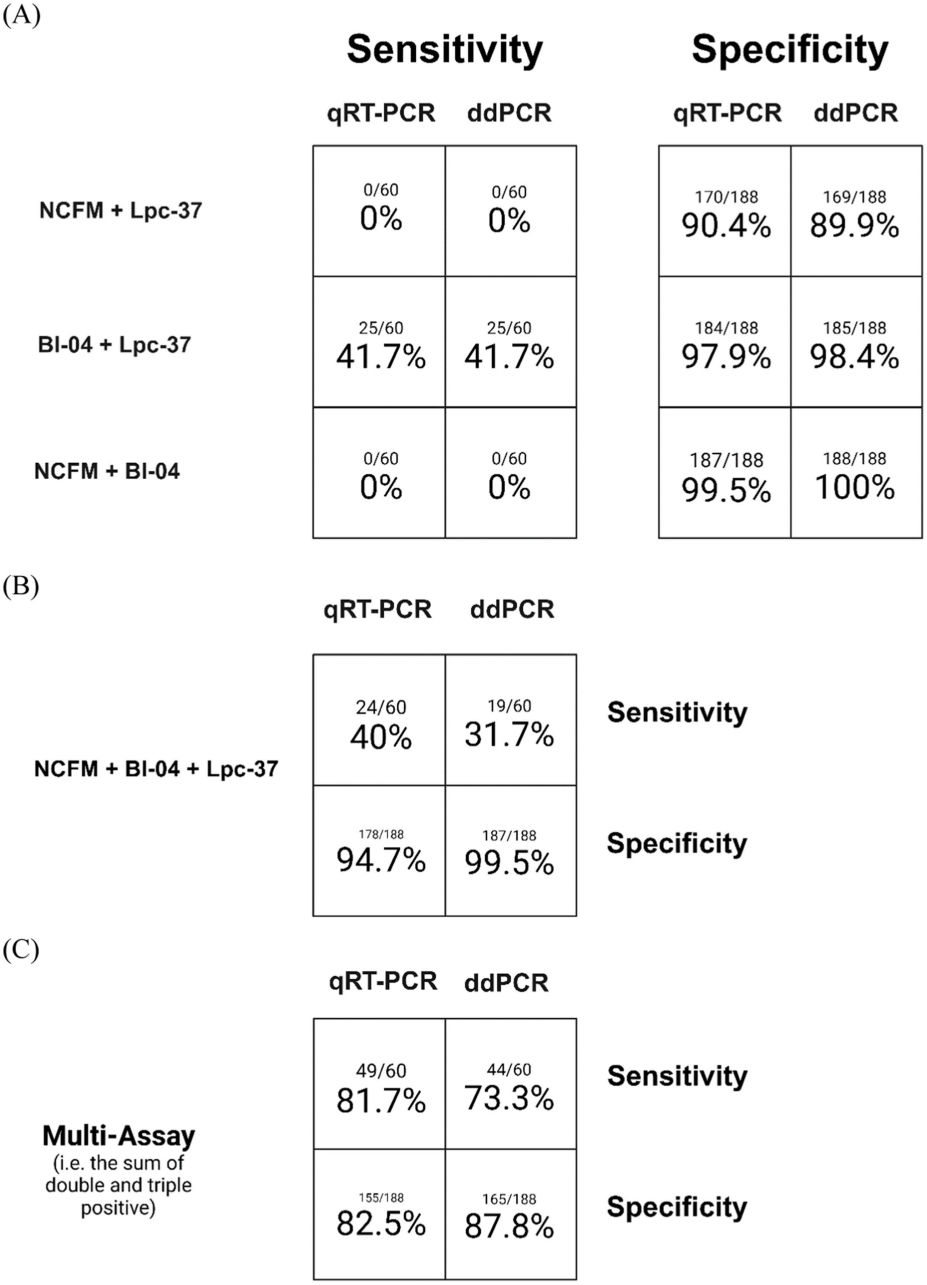
Assay sensitivity (samples observed positive for an assay divided by total samples expected to be positive) and specificity (samples observed to be negative for an assay divided by total samples expected to be negative) for both qRT-PCR and ddPCR. Samples positive for any combination of two assays only **(A)**, for all three assays only **(B)**, and the sum of both multi-assay detections **(C)**.

When looking at the data from a multi-assay perspective (Figure 4) we can see in the sensitivity of these assay combinations there were no samples positive for the NCFM assay as a duplex (Figure 4A) but instead were found as a triplex (Figure 4B). The sum of both the double and triple positive assays is shown in Figure 4C which represents the original strategy for multi-assay detection or any two out of three being considered positive. This is the same analysis that was performed in Airaksinen et al., 2019, but with this current data set.

### Correlation of assays

**Figure 5:**
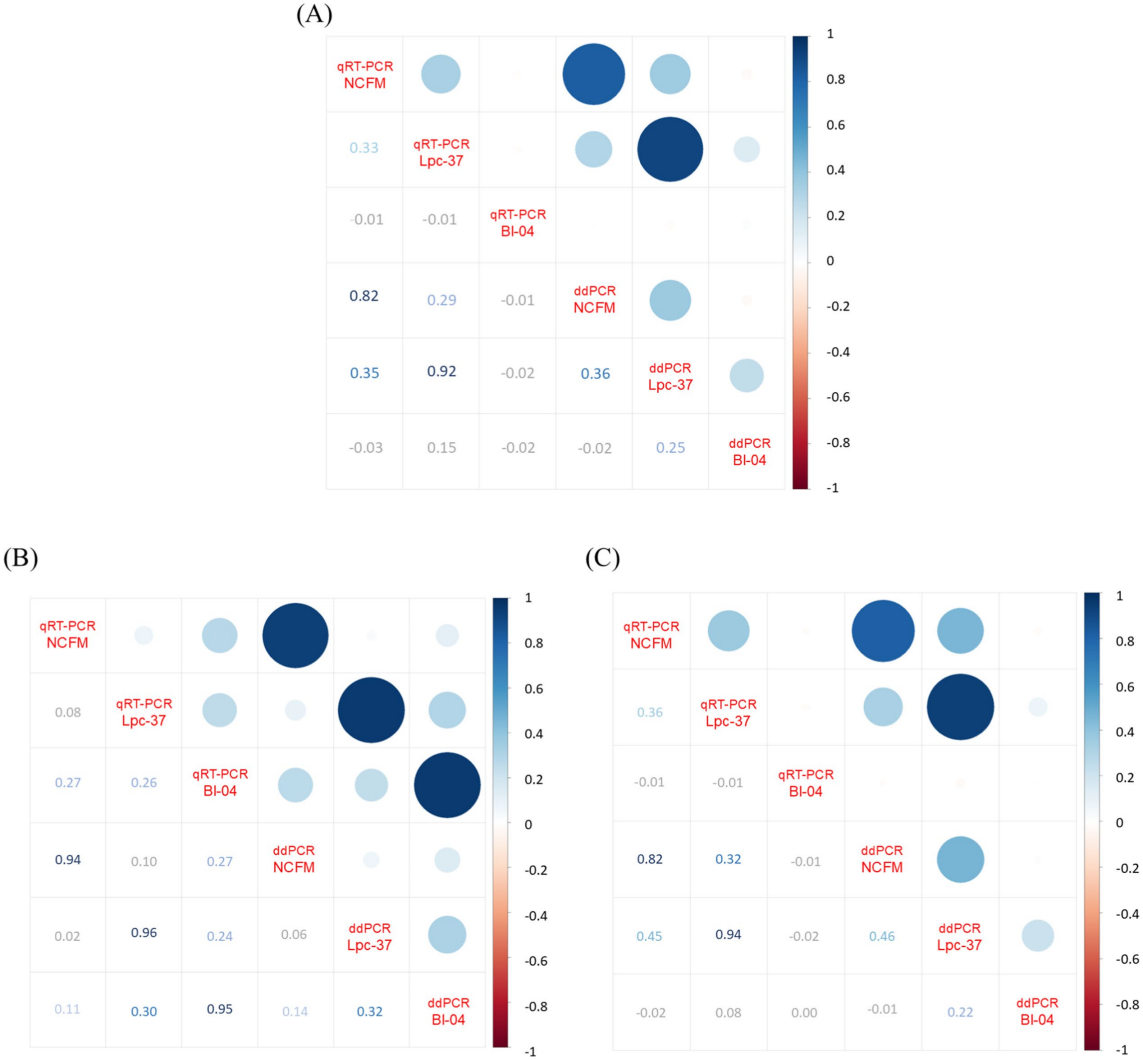
Correlation plot generated by RStudio Build 764 R v4.4.1 package Corrplot v0.92 (using Pearson correlation) from the absolute values of qRT-PCR and ddPCR across all assays per sample, with nulls replaced by lower limits of detection which were set to half the limit of quantification. Panel **(A)** displays the analysis with the whole data set (*n* = 248), **(B)** only the samples from the positive population (*n* = 60) and **(C)** only the samples from the negative population (*n* = 188). Image modified to increase contrast and readability of lower half of all plots.

Finally, we applied a Pearson correlation to the samples across all their values for the three assays and the two methods using an R package called Corrplot version 0.92 (Figure 5). The nulls were replaced with half the value of the lowest standard or lower limit of quantification. Across the entire data set there were no strong negative correlations, and the highest correlations were between the methods: NCFM (0.82), Lpc-94 (0.92) but not for Bl-04 (−0.02) (Figure 5A). Separating the positive (Figure 5B) and negative groups (Figure 5C) we can see that the methods strongly correlate in the positive group: NCFM (0.94), Lpc-37 (0.96), Bl04 (0.95). The negative group had a strong method correlation for NCFM (0.82) and Lpc-37 (0.94) but not Bl-04 (−0.02).

## Discussion

The aim of this work was to compare two modalities of PCR to properly detect probiotic consumption from human clinical trial samples. We compared: the current gold standard in the field of clinical probiotic detection, namely qRT-PCR, and the newest generation of PCR-based technology ddPCR. To that end, we performed an analysis looking at the diagnostic accuracy of 3 PCR assays (NCFM, Bl-04 and Lpc-37) through a sample set from a recent human clinical trial (Airaksinen et al., 2019). We evaluated each assay’s and method’s ability to correctly determine true positives (positive PCR signal from samples in the intervention group) or its sensitivity versus each their ability to correctly determine true negatives (negative PCR signal from samples in the non-intervention group) or its specificity (Yerushalmy, 1947; Glaros and Kline, 1988). Both measures, and indeed any PCR based approach, demand strong primer and probe design and optimization and validation (Bustin et al., 2020). Here we showed that the assays performed well in assay optimization and validation, as each had an expected relationship across a dilution series of DNA from a pure culture (Figure 2). ddPCR demonstrated a lower limit of detection as compared to qRT-PCR, making it a powerful tool for the detection of lower abundance microbial targets. Furthermore, we also demonstrated that the primers and probes used were valid for species-specific and close to strain-specific discrimination by testing against a panel of off target bacteria (Table Sl). Although this validation is limited to the panel described and there could exist microbes that yield a false positive signal. This indicated that assays were performing well according to the parameters we evaluated and were valid to be used on human fecal samples.

Looking at the data overall (Table 2) we can see that in the blinded stage of analysis, meaning that the intervention status of each sample is unknown, there were unusually high PCR signals coming from the negative population. This work focused on this stage of analysis as it reflects how data should be generated and analyzed during the blinded stage of a clinical trial (Schulz et al., 1995). Post-hoc analyses, like per protocol population sub-analyses, can be quite valuable when looking beyond a clinical trial’s primary parameters (Montori and Guyatt, 2001). This type of sub-analysis would then exclude data coming from both those in the intervention group without PCR positive signal from the probiotic assays and those in the non-intervention group that had this unusually high PCR signal.

Having established that the assays were optimized and validated we used three assays to detect a PCR signal from a set of human clinical trial samples, looking first at how each assay performed individually across the diagnostic parameters of sensitivity and specificity (Figure 3). The Lpc-37 assay had the highest sensitivity across both qRT-PCR and ddPCR but also the lowest specificity. Assay design is the tradeoff between being able to detect your target while simultaneously not detecting anything else, and has also proven challenging in other PCR based diagnostic testing (Lemmon and Gardner, 2008). One possible reason might be that the Lpc-37 assay was found to be species specific, and the false positives are likely due to the low-level prevalence of the species *L. paracasei* in the human gut (Pasolli et al., 2020). The NCFM assay had the poorest sensitivity, or true positive rate, across both qRT-PCR (40%) and ddPCR (31.7%). In the context of a human clinical trial the optimal probiotic detection assay would be maximal for both specificity and sensitivity, the Bl-04 assay performed the best in both regards.

Next, we looked at how the specificity and sensitivity values changed if we used a multi-assay criterion (Figure 4). First, we examined the samples positive for any two assays only (meaning that samples positive for all three assays were not counted). Interestingly there were no samples dual positive for any combination including NCFM in the positive group, the dual positive sample were solely Bl-04 with Lpc-37 (Figure 4A). This is likely due to the low sensitivity of NCFM which we described in the prior paragraph. Specificity increased overall when comparing the individual assays to the multi-assay criterion. This stands to reason as the additional criterion for positive discrimination decreases the likelihood of false positives or increases the true negative rate. The triple positive only community was similar to the dual positive only community, indicating that we likely gained no benefit from the addition of a third assay (in this case NCFM). In general, it appears that when adding more than one assay to define a sample as positive there is a drop in sensitivity and an increase in specificity. Looking at the sum of both double and triple positive samples we see similar values across qRT-PCR and ddPCR. There seems to be a general trend for ddPCR to score slightly lower in sensitivity and slightly higher in specificity.

To compare qRT-PCR and ddPCR more directly, we performed a Pearson correlation with the data set (Figure 5). When examining the entire data set between qRT-PCR and ddPCR (Figure 5A), we found that both the NCFM (0.82) and Lpc-37 (0.92) assays correlated positively with each other while the Bl-04 (−0.02) assay did not. We next separated the data set into its true positive (Figure 5B) and negative groups (Figure 5C). All three assays correlate positively across both their methods within the true positive group (NCFM 0.94, Lpc-37 0.96 and Bl-04 0.95). However, in the negatives group we see that only the NCFM (0.82) and Lpc-37 (0.94) assays correlate across methods, which was similar as the entirety of the data set. This is likely due to: the size of the groups (as the negative group was much larger and had an outsized effect on the correlation which then carried over when looking at the whole data set) and the issues with NCFM sensitivity and Lpc-37 specificity.

Taking all this together we can now see that most of the sensitivity and specificity was coming from the Bl-04 assay alone, if we compare the assay individual results versus the multi-assay results. If the study had only used that as its criterion for compliance the overall true positive rate would have been similar as compared to the multi-assay criterion, but the true negative rate would have been improved.

It is of note that while the assay optimization and validation was successful, the diagnostic performance from biological samples varied. Some of the false signal could be attributed to the species and not strain level discrimination of the assays, for bacteria that are commonly found in the human gut. This kind of retrospective view of each assay’s sensitivity and specificity both individually and in combination is a useful tool in deciding whether to continue using any given PCR assay (Broeders et al., 2014; Shehata et al., 2019). This can be the benchmark for further assay development or documenting which assays were appropriate for the sample type and experimental set-up.

Recently, there has been work done with locked nucleic acid (LNA) probes that show the ability to target single nucleotide polymorphisms (SNPs) via qRT-PCR, specifically useful for pulling apart strain differences in closely related probiotics (Shehata et al., 2021). Moving forward, these types of modifications that can increase the accuracy and precision of the PCR signal will be useful. Moreover, it is clear from our data that it is not sufficient to optimize for PCR based criteria, as all the assays described performed well in wetlab testing with pure cultured DNA. It was only by examining their diagnostic performance in biological samples did issues arise. Testing and reporting the PCR-based detection of the bacteria spiked into a fecal sample can be a way forward (Li et al., 2024).

Overall, qRT-PCR and ddPCR performed similarly in the detection of probiotics consumed during an adult human clinical trial, and off the three assays used most of the diagnostic power came from the Bl-04 assay. This suggests that depending on the experimental questions and set-up that the Lpc-37 and NCFM assays may not be appropriate. In the future, ddPCR may be favored for applications where DNA yield is low, such as in clinical trials involving infants or where specificity is prioritized. However, when properly optimized and validated qRT-PCR remains an appropriate method for probiotic detection. ddPCR also carries additional costs (additional equipment, lower throughput) when compared to qRT-PCR necessitating thoughtful consideration when choosing one over the other (Zhang et al., 2024). Advancements in PCR assay optimization and validation can also be made to increase the value of the data generated from human clinical trials involving probiotics.

## Data Availability

The raw data supporting the conclusions of this article will be made available by the authors, without undue reservation.
