# Supplementary material for: Comparison of qRT-PCR and ddPCR for multi-strain probiotic detection after a randomized human clinical trial
Source: Front Microbiol. 2025 Apr 28;16:1579797. doi: 10.3389/fmicb.2025.1579797 (PMC12066471; doi:10.3389/fmicb.2025.1579797)
Supplement: Supplementary file 1 [file Table_1.pdf]

Supplementary material

Assay	Bacterial Strains	Detection (yes/no)
NCFM		
	Lactobacillus acidophilus La-14	No
	Lactobacillus acidophilus La4356	Yes
	Lacticaseibacillus rhamnosus ATCC 53103	No
	Bacteroides fragilis ATCC 25285	No
	Faecalibacterium duncaniae DSM 107838	No
	Ligilactobacillus salivarius Ls-33	No
	Blautia producta DSM 2950	No
	Bifidobacterium infantis Bi-26	No
	Bifidobacterium animalis subsp. lactis Bi-07	No
	Enterocloster bolteae DSM 15670	No
	Clostridium perfringens ATCC 13124	No
	Escherichia coli K-88	No
	Limosilactobacillus fermentum DSM 20052	No
	Lactobacillus farciminis ATCC 29644	No
	ZymoBIOMICS Microbial Community DNA Standard D6306	No
Lpc-37		
	Bacteroides fragilis ATCC 25285	No
	Faecalibacterium duncaniae DSM 107838	No
	Lacticaseibacillus casei Lc20	No
	Lacticaseibacillus casei Lc11	No
	Lacticaseibacillus rhamnosus ATCC 53103	No
	Ligilactobacillus salivarius Ls-33	No
	Lactiplantibacillus plantarum 11715	No
	Levilactobacillus brevis LBR-35	No
	Limosilactobacillus fermentum DSM 20052	No
	Lactobacillus acidophilus NCFM	No
	Lactobacillus helveticus 4451	No
	Bifidobacterium infantis Bi-26	No
	Bifidobacterium animalis subsp. lactis Bl-04	No
	Bifidobacterium animalis subsp. lactis Bi-07	No
	Bifidobacterium animalis subsp. lactis HN019	No
	ZymoBIOMICS Microbial Community DNA Standard D6306	No
Bl-04		
	Bifidobacterium animalis subsp. lactis Bi-07	Yes
	Bifidobacterium animalis subsp. lactis B420	No
	Bifidobacterium animalis subsp. lactis HN019	No
	Bifidobacterium infantis Bi-26	No
	Bifidobacterium adolescentis DSM 20083	No
	Bifidobacterium breve Bb-03	No
	Bifidobacterium saeculare DSM 6531	No
	Faecalibacterium duncaniae DSM 107838	No
	ZymoBIOMICS Microbial Community DNA Standard D6306	No

Table S1. Panel of strains used during assay validation in both qRT-PCR and ddPCR for each assay. ZymoBIOMICS Microbioal Community DNA Standard D6306 is made up of the following species: *Pseudomonas aeruginosa, Escherichia coli, Salmonella enterica, Lactobacillus fermentum, Enterococcus faecalis, Staphylococcus aureus, Listeria monocytogenes, Bacillus subtilis, Saccharomyces cerevisiae* (yeast) and *Cryptococcus neoformans* (yeast).
